# Metazoan diversification characterized by elevated rates of evolution during the middle Ediacaran to earliest Cambrian

**DOI:** 10.1126/sciadv.aeh7099

**Published:** 2026-10-30

**Authors:** Zhiqing Guo, Thomas J. Roper-Smith, James W. Clark, Jordi Paps, Ruolin Wu, Davide Pisani, Philip C. J. Donoghue

**Affiliations:** ^1^ Bristol Palaeobiology Group, Schools of Earth and Biological Sciences, University of Bristol, Life Sciences Building, Bristol BS8 1TQ, UK.; ^2^ Oxford University Museum of Natural History, South Parks Road, Oxford OX1 3PW, UK.; ^3^ Centre for Evolution, Department of Life Sciences, University of Bath, Bath BA2 7AY, UK.

## Abstract

To test causal hypotheses for the emergence of animal body plans, we analyze rates of molecular and phenotypic evolution across Opisthokonta, focusing on divergences within the Tonian-Ordovician interval. Both molecular and phenotypic rates increase through the Ediacaran, peaking in an Ediacaran and earliest Cambrian interval before declining thereafter. Partitioning phenotypic characters into categories relating to development, feeding, fossilization potential, locomotion, and nervous systems reveals distinct temporal patterns, with developmental traits contributing disproportionately to elevated rates in the metazoan stem lineage. Rate patterns show a temporal lag between the ecological changes integral to hypotheses of developmental evolution and the Cambrian agronomic, substrate, and information revolutions and earlier peaks in the evolution of the associated phenotypic characters. These patterns are largely independent of the timescale used. Overall, our results indicate that the primary phase of elevated evolutionary novelty occurred during the Ediacaran and culminated in the late Ediacaran-earliest Cambrian, providing the foundation for later ecological and taxonomic innovation.

## INTRODUCTION

The emergence of animals represents one of the most profound evolutionary transitions in Earth history, laying the foundations of modern biodiversity (*1*) and precipitating formative changes to the marine environment, including the aeration of sediments (*2*) and the water column (*3*) and accelerating the biological pump (*4*). Nevertheless, the timing, tempo, and mode of this evolutionary episode remain contentious. While there is a fossil record of unequivocal animals that extends deep into the Ediacaran (*5*, *6*), the fossil record of bilaterians begins abruptly at or around the Ediacaran-Cambrian boundary [∼538.8 million years ago (Ma)] (*7*–*10*). However, molecular timescales support an earlier origin of animals and bilaterians (*11*–*16*). This discrepancy between divergence times and first fossil occurrences continues to fuel debate over whether the Cambrian reflects a major diversification and/or a pulse of evolutionary innovation (*17*, *18*) or merely an increase in the fossilization potential of early animals (*7*, *19*). Candidates for drivers of biological innovation are not mutually exclusive, including developmental evolution (*20*), the environment (*21*), motility, and feeding in the Cambrian Substrate Revolution (*22*) and the Cambrian Information Revolution (*23*, *24*). Meanwhile, the parallel evolution of mineralized skeletons undoubtedly led to an increase in the fossilization potential of early animals (*25*). However, the association between these innovations and the phenomena they seek to explain are often little more than anecdotal, with few notable attempts to quantitatively characterize them or the Cambrian explosion hypothesis more generally (*21*).

The few attempts to characterize rates of evolution associated with early animal evolution include Aris-Brosou and Yang (*26*) who examined rates of molecular evolution in just two genes, finding evidence of accelerated rates associated with the divergence of protostomes and deuterostomes. Molecular and phenotypic rates have been best characterized for arthropods (*27*) in which fast early rates are observed across all lineages during the Cambrian, followed by much lower rates post-Cambrian. The extent to which arthropods are representative of animals more broadly is unclear, and so we took a broader perspective, analyzing rates of molecular and phenotypic evolution across Metazoa and including opisthokont outgroups on the basis of (i) a molecular dataset from (*11*), which is composed of 203 protein genes from 66 taxa, including 4 holozoan outgroups, 10 nonbilaterians, and 43 bilaterians, including representatives of 16 animal phyla, and (ii) a phenotypic dataset modified from (*28*) that was subsampled taxonomically to match the taxon set in the molecular dataset. We consider drivers of innovation and interpretations of the fossil record by estimating rates of evolution in subsets of phenotypic characters that relate to these hypotheses. Inferences of evolutionary rate are contingent on the evolutionary timescale. We describe our results on the basis of the most recent molecular timescale (*11*), which estimates an early-middle Ediacaran origin of animals, a middle-late Ediacaran origin of bilaterians, and a late Ediacaran-early Cambrian origin of the bilaterian phyla. However, we also consider the impact of alternative timescales, including (i) dos Reis *et al.* (*12*), who estimated a Tonian-Cryogenian origin of animals, late Cryogenian-early Ediacaran origin of bilaterians, and deep Ediacaran origins of the bilaterian phyla, and (ii) Budd and Mann (*17*), who inferred a middle Ediacaran origin of animals, latest Ediacaran origin of bilaterians, and Cambrian origin of bilaterian phyla.

## RESULTS

### Molecular and phenotypic evolutionary rates

We estimated rates of molecular evolution in MCMCtree (*29*) using the dataset of Carlisle *et al.* (*11*). After normalizing branch-specific rates by the mean clock rate, the resulting dimensionless relative molecular rates ranged from 0.033 to 2.093, where values >1 indicate faster-than-average evolution and values <1 indicate slower-than-average branches. Elevated rates appear to be concentrated on deep branches within animal phylogeny and diminish toward the tips of the tree (Fig. 1A). Markedly accelerated rates (defined as branch-specific rates exceeding one standard deviation above the mean background rate distribution; threshold = 1.13; see Materials and Methods) occur on the stem lineages of Eumetazoa, Parahoxozoa, Deuterostomia, Bilateria, Nephrozoa, Protostomia, and Spiralia (fig. S2). The highest relative rate (2.093) occurs on the bilaterian stem lineage. At the phylum level, initial relative rates commonly exceeded 1. These rates then gradually decline in more derived lineages, with values falling below 1 (Fig. 1, A and B).

**Fig. 1.**
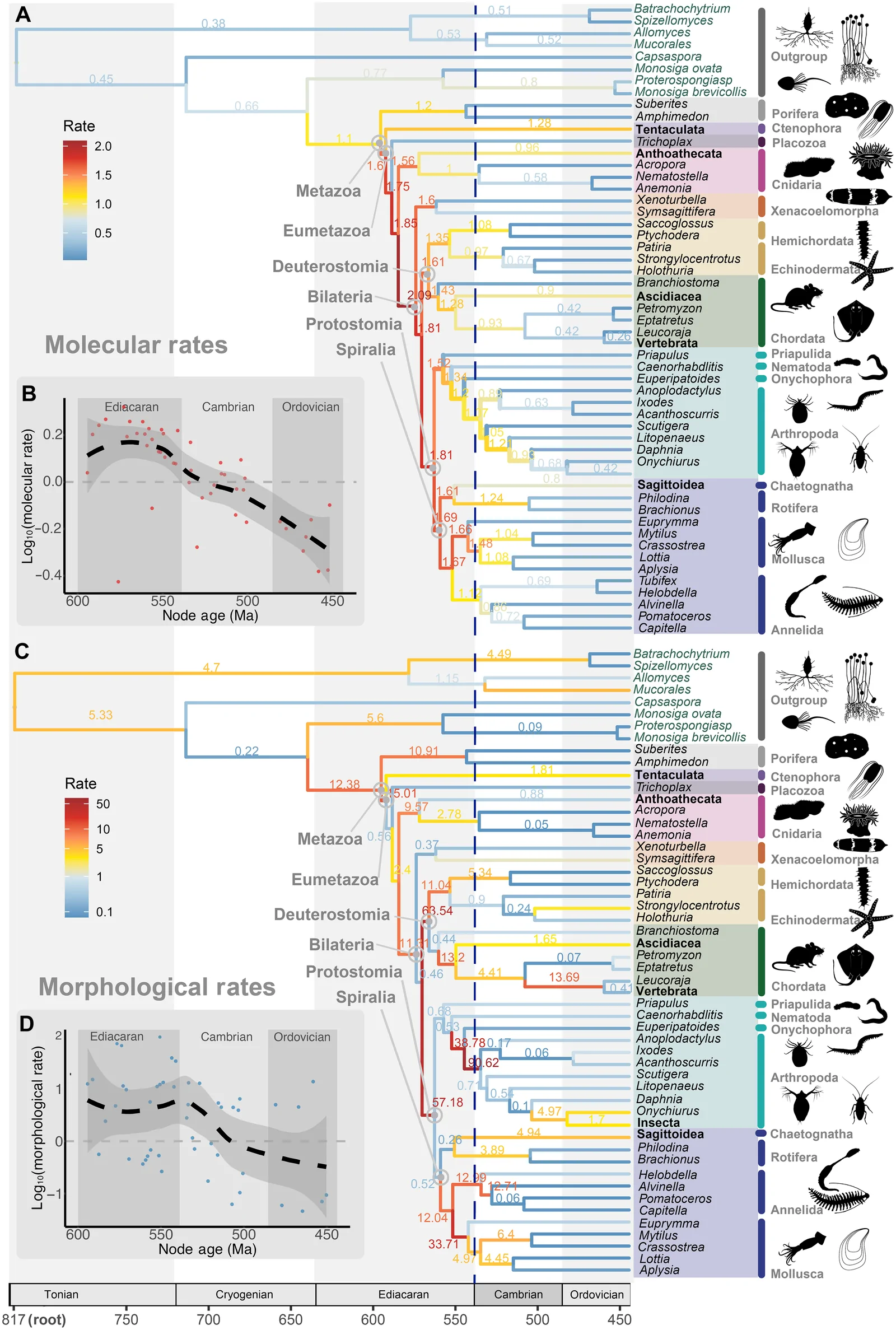
Evolutionary rate variation across deep metazoan branches. (**A**) The phylogenetic tree illustrates variations in relative molecular substitution rates across different animal taxa, represented by a blue-to-red gradient (blue = slower; red = faster). Rates are relative to the mean branch rate estimated under the relaxed clock model [0.166 substitutions per site per Myr (subs/site/Myr)]. For clarity and because of uncertainty in rate estimation, values for terminal branches are not displayed. (**B**) Temporal trends of relative molecular evolutionary rates. Log_10_-transformed relative rates (*y* axis) are plotted against node age (Ma; *x* axis). Red points and the dashed line represent molecular rates and LOESS regression curve, respectively. The analysis includes branches from the Ediacaran, Cambrian, and Ordovician periods. A dashed gray line at *y* = 0 indicates the threshold between accelerated (>0) and slower (<0) relative rates. (**C**) The phylogenetic tree illustrates variations in relative morphological rates across different animal taxa. Phylogenetic tree illustrating variations in relative morphological substitution rates across internal branches up to the end of the Ordovician period. This analysis is based on 2232 phenotypic characters using a relaxed morphological clock model. Rates are relative to the mean branch rate estimated under the relaxed clock model [0.000585 substitutions per character per Myr (subs/chara/Myr)]. (**D**) Temporal trends of relative morphological evolutionary rates. Blue points and the dashed blue line represent morphological rates and their LOESS regression curve, respectively. Shaded areas indicate the 95% confidence intervals (CIs) for each LOESS curve. Full evolutionary rate results are provided in fig. S1.

Markedly accelerated phenotypic rates (>12.10; Figs. 2F and 3F) occurred predominantly within a 10-million-year interval immediately preceding the Cambrian on the stem lineages of Metazoa, Eumetazoa, Deuterostomia, and Protostomia (Fig. 1C). Elevated rates also marked the early diversification of several animal phyla, with markedly accelerated rates occurring on the stem branches of Arthropoda, Annelida, and Mollusca, and elevated rates on Porifera, Rotifera, Chaetognatha, Hemichordata, Cnidaria, and Ctenophora. Among them, Arthropoda consistently exhibited the highest rates of phenotypic evolution. In contrast, clades such as Xenacoelomorpha, Echinodermata, Priapulida, and Chordata displayed comparatively low rates of phenotypic evolution.

**Fig. 2.**
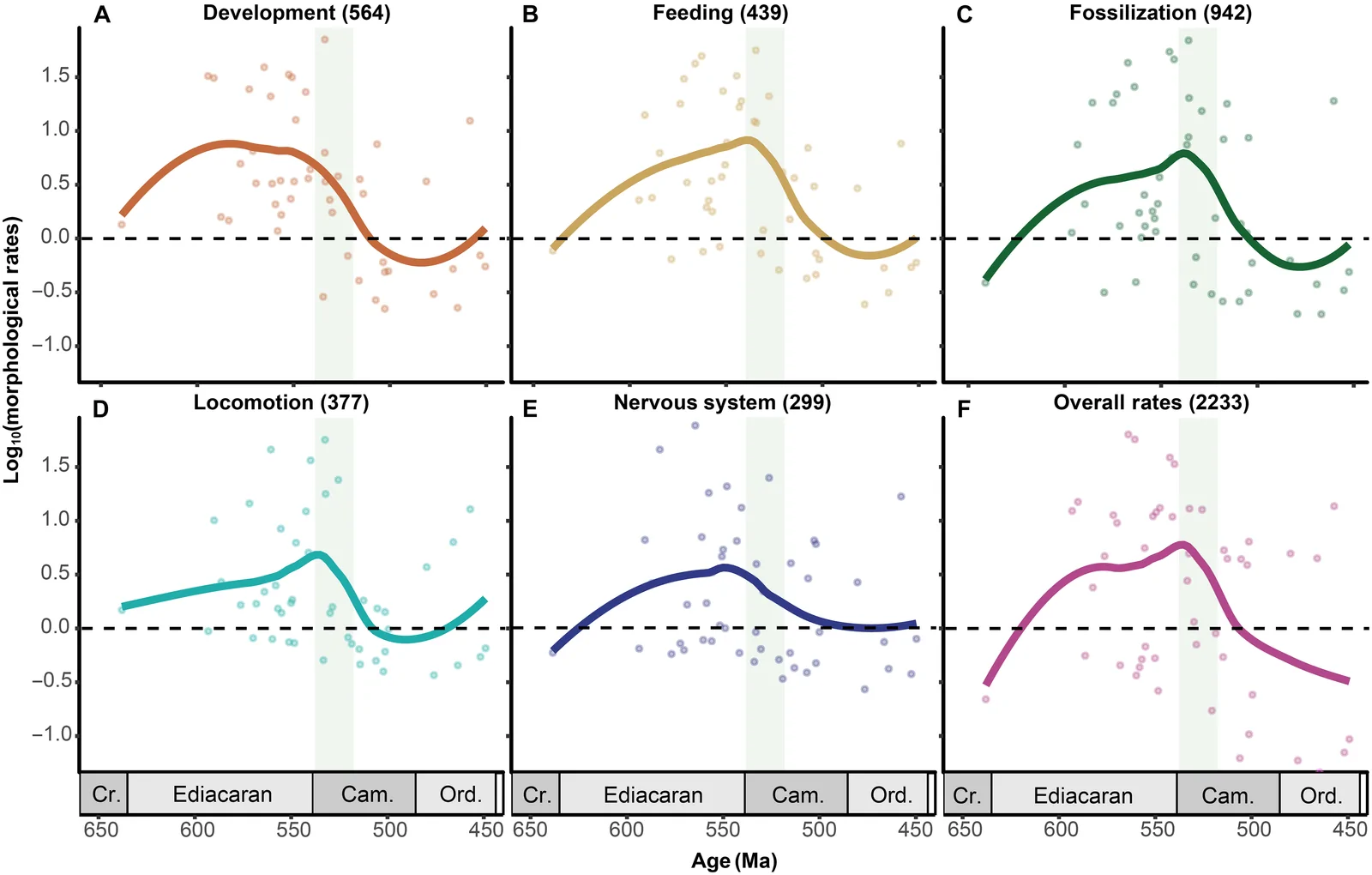
Temporal dynamics of morphological evolutionary rates for different functional categories. Log-transformed relative morphological rates (*y* axis) for individual branches are plotted against geologic time (*x* axis) for six-character categories: (**A**) development, (**B**) feeding, (**C**) fossilization, (**D**) locomotion, (**E**) nervous system, and (**F**) overall. Rates are presented for branches falling within the Ediacaran, Cambrian, and Ordovician periods. A LOESS regression curve (solid line) is fitted to the data points to illustrate general trends. The dashed horizontal line at 0 indicates the threshold between “accelerated” (rates >0) and “slower” (rates <0) relative rates. The shaded green area represents the Cambrian explosion time interval.

**Fig. 3.**
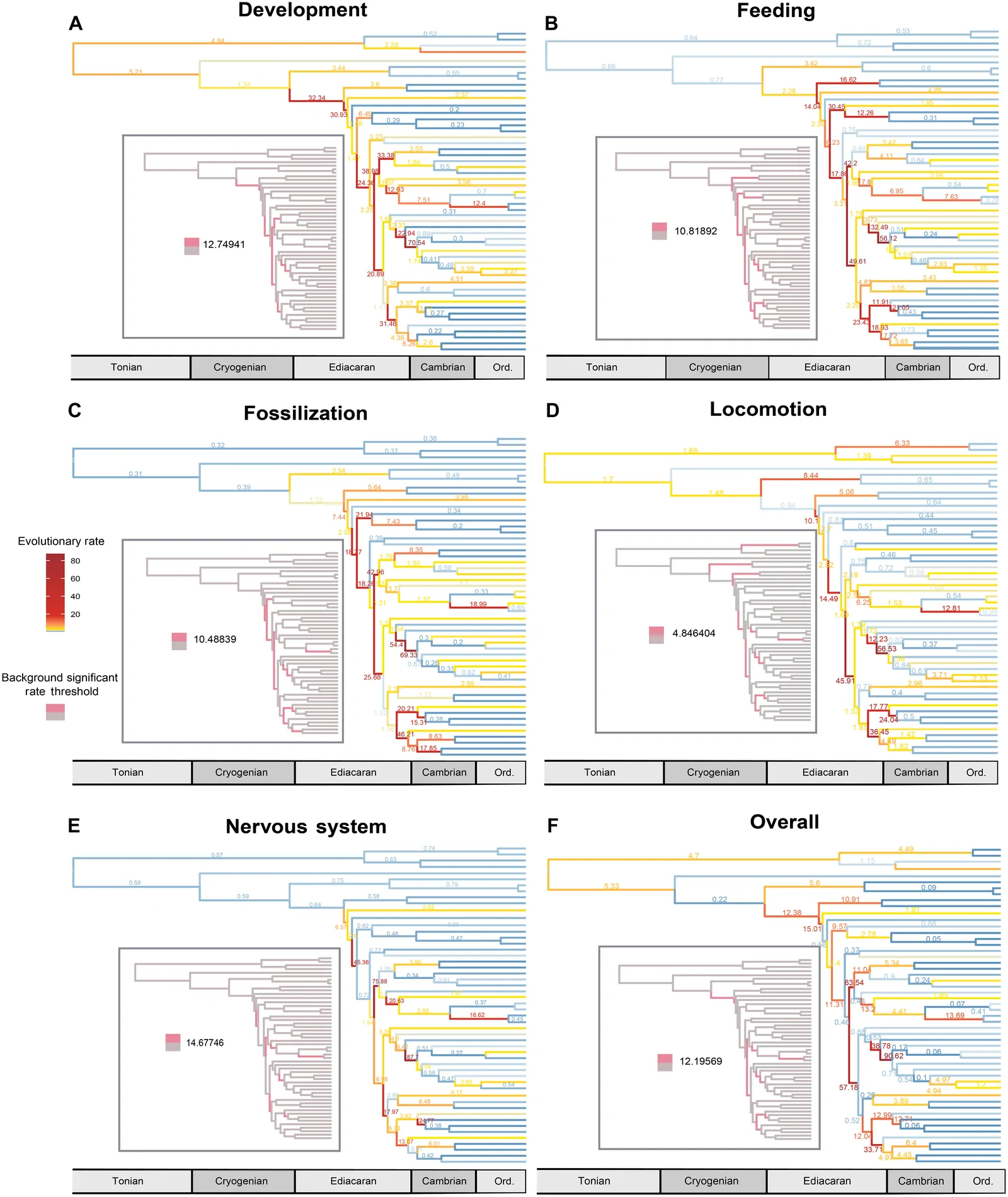
Phylogenetic distribution of morphological rates and marked shifts in functional categories. The phylogenetic trees illustrate variations in relative morphological rates across early animal taxa, with branches colored according to their respective functional categories. Each panel (A to F) represents a different character category: (**A**) development, (**B**) feeding, (**C**) fossilization, (**D**) locomotion, (**E**) nervous system, and (**F**) overall. Branches exhibiting marked rate increases are highlighted in pink, with specific thresholds for each category as follows: development (>12.74941), feeding (>10.81892), fossilization (>10.48839), locomotion (>4.846404), nervous system (>14.67746), and overall (>12.19569). Thresholds were calculated separately for each character category on the basis of the mean and standard deviation of its background rate distribution (see Materials and Methods). Branches with nonmarked rate shifts are colored grey.

Given that both molecular and phenotypic data exhibit peaks in the rate of evolution during the Ediacaran and earliest Cambrian, we explored whether these rates were correlated (fig. S3). Molecular evolutionary rates obtained from relaxed-clock analyses were converted to relative rates [branch-specific rate divided by the estimated base clock rate: 0.1655 substitutions per site per million years (Myr)] and then log_10_ transformed to make them directly comparable to their morphological counterparts (see Materials and Methods). Locally estimated scatterplot smoothing (LOESS) regression was applied to both morphological and molecular relative rates to assess their temporal trends through the Ediacaran and Phanerozoic.

The relative rates between molecular evolution and phenotypic evolution are not well correlated (linear regression: *y* = 0.251 + 0.0591*x*, *R*^2^ = 0.07; fig. S3). Morphological rates exhibit more variability, characterized by a prominent peak and subsequent decline (Fig. 1D). In contrast, molecular rates are less variable, with a minor rise in the early Ediacaran that peaks in the middle Ediacaran, followed by a long-term decline extending through the Cambrian and into the Ordovician (Fig. 1B). To quantitatively assess the correlation between these two evolutionary scales, we compared log_10_-transformed morphological rates and log_10_-transformed molecular rates using linear regression. The fitted relationship was weak (*R*^2^ = 0.07; fig. S3). To account for potential temporal autocorrelation in the rate time series, we additionally analyzed the data using ARIMA-based models. The 10-Myr binning procedure produced 27 temporal bins. The number of branches contributing to individual bins varied, with some older bins represented by only one or two branches (table S4). The raw cross-correlation function (CCF) (fig. S5) provided an exploratory view of the temporal correspondence between binned molecular and morphological rates. However, because both rate series may contain a temporal structure, we placed greater emphasis on the prewhitened CCF (fig. S5). The molecular rate series was best described by an ARIMA(0,1,1) model, which was used to prewhiten the molecular series and to apply the equivalent filter to the morphological series.

The prewhitened CCF (fig. S5) did not provide strong evidence for a consistent lead-lag relationship between molecular and morphological rates. In the ARIMAX analysis (tables S3 and S4), molecular rates did not significantly predict morphological rates after binning and accounting for the selected error structure. The molecular-rate coefficient was positive but nonsignificant (β = 0.273, SE = 0.319, *z* = 0.855, *P* = 0.393) (table S4). The ARIMAX model (table S4) was also less supported than the null ARIMA model (table S4) on the basis of Akaike information criterion (AIC), with AIC increasing from 98.94 in the null model to 101.20 in the model including molecular rates. Together, the branch-level regressions and time-series analyses provide limited (if any) support for an association between molecular and phenotypic rates in the sampled datasets under the models examined.

### Partitioned rates of phenotypic evolution

To gain a better understanding of the nature of characters that change on branches that exhibit accelerated rates of evolution, we organized the phenotypic characters into five nonmutually exclusive categories that relate to hypotheses of biological innovation associated with animal diversification or interpretations of the fossil record. These groups include characters associated with the nervous system (299 characters), which we use to characterize the hypothesis of a Cambrian Information Revolution (*23*, *24*); feeding (439 characters) and locomotion (377 characters), which, together, relate to the Cambrian Substrate (*22*) and Agronomic revolutions (*30*, *31*); and fossilizable characteristics (942 characters) to characterize the hypothesis that the Cambrian Explosion reflects an increase in the fossilization potential of animals (*19*) and characters that relate to development and reproduction (564 characters), which we use to characterize the role of developmental evolution in animal diversification (*20*). All five categories exhibit variation in the rate of evolution, but a LOESS regression shows increasing rates through the Ediacaran that peak in the middle Ediacaran (development and nervous system characters; Fig. 2, A and E) or in the latest Ediacaran-earliest Cambrian interval (feeding, fossilization, locomotion, and overall rates; Fig. 2, B, C, D, and F, respectively), followed by a sustained decline extending into the mid-Cambrian and beyond when the LOESS rates dropped below zero. These similarities notwithstanding, each of the character partitions exhibits differences in their LOESS regression lines. Characters associated with development exhibit the earliest peak in rates in the early Ediacaran, which is sustained through the late Ediacaran (Fig. 2A); these character state changes occur on the stem lineages of Metazoa, Eumetazoa, Bilateria, Deuterostomia, Protostomia, Lophotrochozoa, Arthropoda, and Ambulacraria (Fig. 3A), and they must contribute disproportionately to the early increase in overall phenotypic rates (Fig. 2F). Characters related to feeding (Fig. 2B) and characters with high fossilization potential (Fig. 3C) also show high early rates, but they increase in rate more gradually and peak at the end of the Ediacaran. High evolutionary rates among characters associated with feeding occur on the stem lineages to Porifera, Eumetazoa, Cnidaria, Bilateria, Deuterostomia, Protostomia, and Lophotrochozoa (Fig. 3B), while faster rates among fossilization-related characters occur on the stem lineages to Parahoxozoa, Cnidaria, Bilateria, Deuterostomia, Protostomia, Mollusca, and Annelida (Fig. 3C). Characters associated with locomotion (Fig. 2D) and the nervous system (Fig. 2E) exhibit the lowest and slowest increase in rates among the character partitions, with less prominent peak rates at the end-Ediacaran and early Cambrian, respectively. Branches that exhibit accelerated rates of evolution among locomotion-related characters occur on the stem lineages to Porifera, Eumetazoa, Bilateria, Protostomia, and Lophotrochozoa (Fig. 3C) and, for nervous system–related characters, on the stem lineages to Planulozoa, Deuterostomia, Chordata, and Spiralia.

### Robustness to competing evolutionary timescales

Our estimates of the rates of phenotypic and molecular evolution are dependent, inevitably, on the timescale on which they are estimated. Given that the timescale of early animal evolution is contested (*11*–*14*, *16*, *17*, *32*), we explored the impact on rate estimates of alternative timescales. We considered the timescale from dos Reis (*12*), which estimates a Tonian origin of Metazoa and a Cryogenian-Ediacaran origin of Bilateria, and a timescale based on Budd and Mann (*17*), who estimated a late Ediacaran origin of Metazoa and Bilateria (565 and 545 Ma, respectively). Budd and Mann (*17*) did not estimate an overall timescale, and so we used their estimates for the age of Metazoa, Eumetazoa, Bilateria, and Protostomia to recalibrate the molecular clock analysis of Carlisle *et al.* (*11*), from which we retained all other clade age estimates.

Across all three alternative evolutionary timescales, estimates of phenotypic evolutionary rates exhibit broadly congruent temporal patterns. Rates decline through the early Ediacaran and reach a minimum in the mid-Ediacaran, after which they increase gradually to a peak close at the Ediacaran-Cambrian transition (Fig. 4). This peak is followed by a precipitous decline through the early to mid-Cambrian, after which rates continue to decrease but at a reduced pace (Fig. 4). Through the Ordovician, all three timescales show a further decline, although some differences emerge: Rates under the timescale from Carlisle *et al.* (*11*) continue to decrease, and those under the timescale from dos Reis *et al.* (*12*) show a slight rebound, whereas the calibration of Budd and Mann (*17*) yields a more pronounced recovery. Nevertheless, despite these quantitative differences, the qualitative pattern of phenotypic rates is highly consistent across timescales (Fig. 4).

**Fig. 4.**
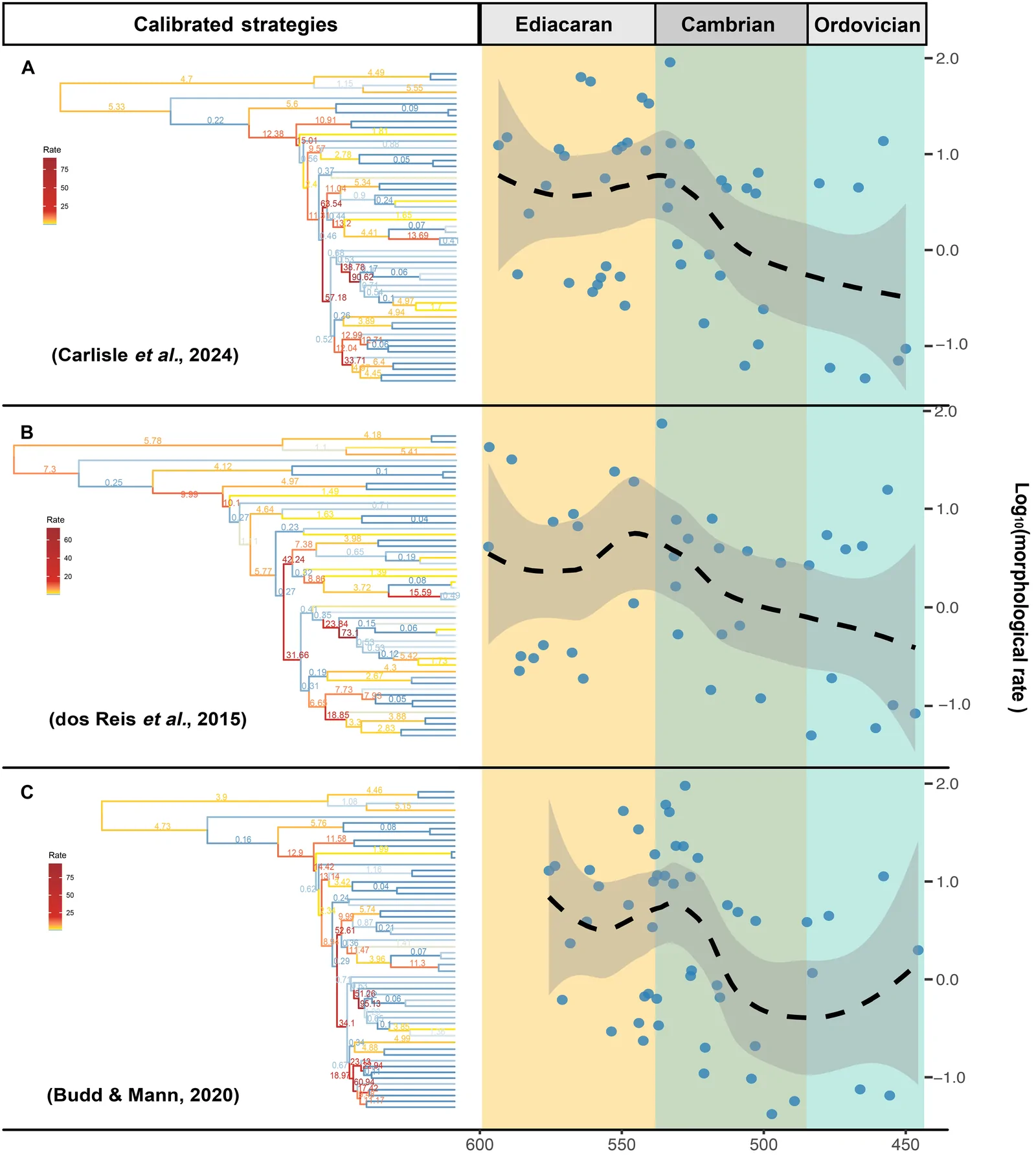
Robustness of morphological evolutionary rate trajectories across divergence-time calibration strategies from the Ediacaran to the Ordovician. (**A** to **C**) Molecular evolutionary rate estimates under three alternative divergence-time calibration strategies: (A) the main timescale following Carlisle *et al.* (*11*), (B) the Tonian-Cryogenian scenario following dos Reis *et al.* (*12*), and (C) the late Ediacaran scenario following Budd and Mann (*17*). For each calibration strategy, the left subpanel shows the dated phylogeny with branches colored by log_10_-transformed morphological rate (warmer = faster). The right subpanel plots branch-wise log_10_-transformed rates (*y* axis) against branch age (Ma; *x* axis), with a LOESS smooth (black dashed) and 95% confidence band (gray). Background shading marks geological periods (Ediacaran, Cambrian, and Ordovician). All panels use the same topology and morphological dataset; only node-age calibrations differ among strategies.

Patterns of molecular evolutionary rates are more sensitive to the choice of timescale. Under timescales from Carlisle *et al.* (*11*) and dos Reis *et al.* (*12*), molecular rates increase from the early Ediacaran to a peak in the mid- to late Ediacaran, followed by a sustained decline through the Cambrian and into the Ordovician, with only a minor increase in the early Cambrian in the timescale from dos Reis *et al.* (*12*) (Fig. 5). In contrast, the calibrations of Budd and Mann (*17*) produce a distinct pattern, with an initial modest decline, a subsequent increase late in the Ediacaran culminating in an early Cambrian peak, and then a prolonged decrease punctuated by a modest Ordovician recovery. Thus, molecular rates show greater variation among timescales than phenotypic rates (Figs. 4 and 5).

**Fig. 5.**
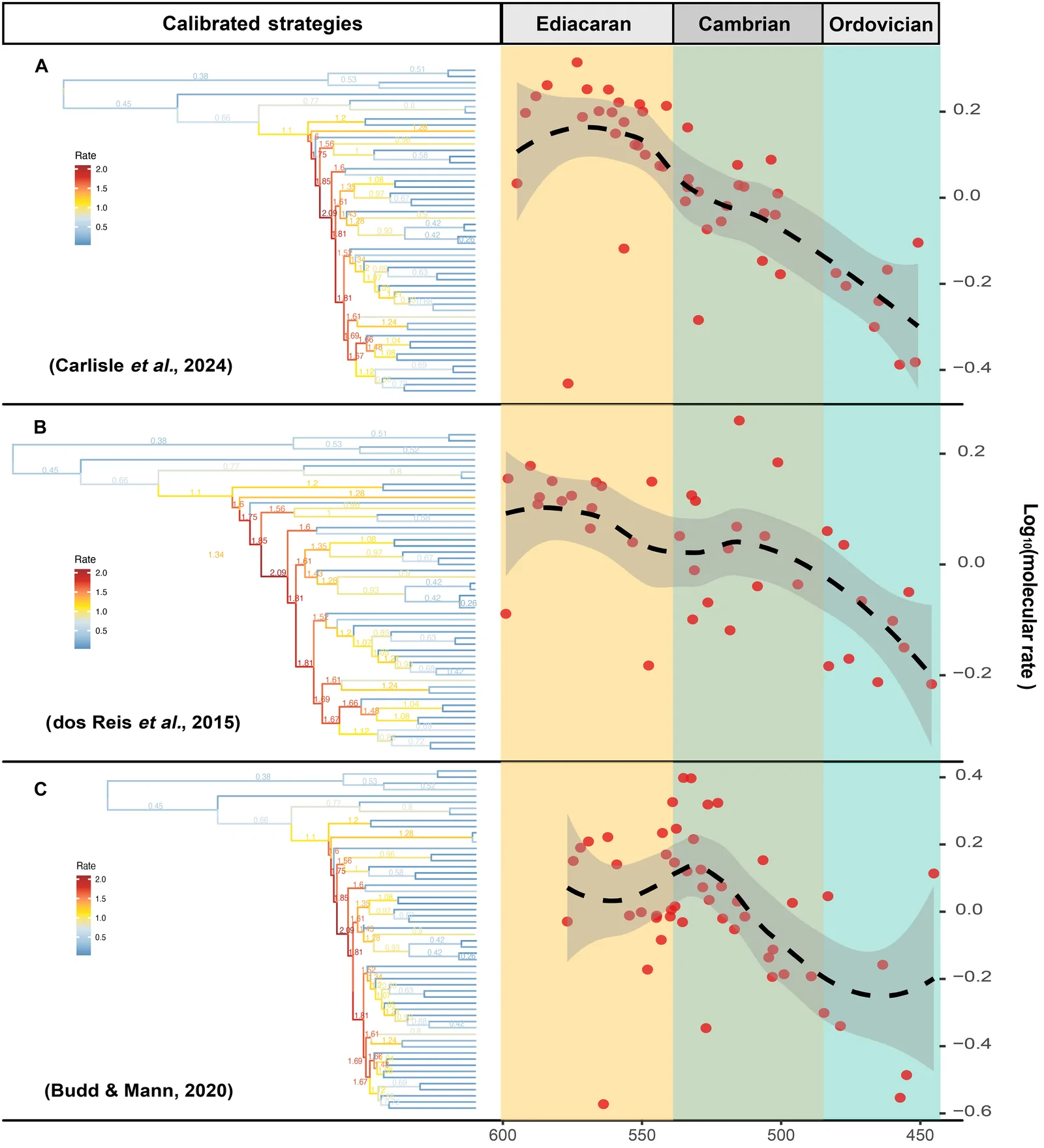
Robustness of molecular evolutionary rate trajectories across divergence-time calibration strategies from the Ediacaran to the Ordovician. (**A** to **C**) Molecular evolutionary rate estimates under three alternative divergence-time calibration strategies: (A) the main timescale following Carlisle *et al.* (*11*), (B) the Tonian-Cryogenian scenario following dos Reis *et al.* (*12*), and (C) the late Ediacaran scenario following Budd and Mann (*17*). For each calibration strategy, the left subpanel shows the dated phylogeny with branches colored by log_10_-transformed molecular rate (warmer = faster). The right subpanel plots branch-wise log_10_-transformed rates (*y* axis) against branch age (Ma; *x* axis), with a LOESS smooth (black dashed) and 95% confidence band (gray). Background shading marks geological periods (Ediacaran, Cambrian, and Ordovician). All panels use the same topology and morphological dataset; only node-age calibrations differ among strategies.

## DISCUSSION

### Patterns of evolutionary rates

Our analyses indicate that, overall, molecular and phenotypic rates peak during the Ediacaran-earliest Cambrian interval, followed by declines through the Cambrian and Ordovician. Molecular rates peak in the middle Ediacaran, whereas phenotypic rates peak in the late Ediacaran-earliest Cambrian. The distribution of elevated rates also differs between the molecular and phenotypic datasets and among clades. The highest rates of molecular evolution occur along the backbone of the phylogeny, from the ancestral crown-metazoan to crown-protostomes and crown-deuterostomes; the component clades of bilaterians and the phyla exhibit markedly lower rates of molecular evolution. Meanwhile, the highest rates of phenotypic evolution are associated with the metazoan stem lineage and crown group, Porifera, crown-Nephrozoa, and clades within Ecdysozoa and Lophotrochozoa. Within the phenotypic dataset, there are also marked differences in the rate of evolution among different classes of characters, with developmental characters peaking the earliest in the middle Ediacaran (Fig. 2A), with characters linked to feeding (Fig. 2B), fossilization (Fig. 2C), and locomotion (Fig. 2D), reflecting accelerated changes earlier or later in phylogeny (Fig. 3, A to D), respectively. These patterns, but not their timing, remain the same regardless of the timescale used (Fig. 4).

These results compare favorably with the few previous attempts to characterize rates of evolution in early animal phylogeny while substantially extending their phylogenetic and temporal scope (*27*, *33*). Previous studies have largely focused on localized case studies within restricted fossil windows, particularly Cambrian arthropods and trilobites. For example, Lee *et al.* (*27*) showed exceptionally elevated phenotypic and genomic rates along early arthropod branches during the Cambrian explosion on the basis of a combined morphological and molecular relaxed-clock framework. Likewise, Paterson *et al.* (*33*), using Bayesian tip dating of a comprehensive trilobite dataset, demonstrated that evolutionary rates were already near background Phanerozoic levels by ∼521 Ma, implying that the principal burst of innovation had largely concluded by the earliest conventional Cambrian fossil record.

Using an 18*S* relaxed clock across Metazoa, Aris-Brosou and Yang (*26*) likewise recovered the highest rates of molecular evolution in the Ediacaran, followed by a decline, although they noted that the timing and magnitude of the slowdown are sensitive to model choice and priors in a single-gene setting. Lee *et al.* (*27*) demonstrated markedly elevated phenotypic and genomic rates within the deepest branches of arthropod phylogeny, followed by a post-Cambrian decline. In our study, phenotypic rates peak in the latest Ediacaran-earliest Cambrian interval (Fig. 1, C and D), but molecular rates are elevated on deep stems from the middle Ediacaran, reducing before the Cambrian (Figs. 1, A and B, and 5A). Notably, the branch leading to Arthropoda shows the highest phenotypic rate in our tree (90.62), reproducing the accelerated rates reported by Lee *et al.* (*27*). Corroboration of our results with these previous studies provides some confidence that they are recovering a faithful record of evolutionary rates in early animal evolution. However, our results suggest that the pattern observed in arthropods by Lee *et al.* (*27*) is not representative of animals, more generally, which exhibit more heterogeneous rates across the tree, associated with the deep stems of animal phylogeny and specific clades, including Porifera and Nephrozoa. Perhaps, the most notable observation from all three studies is not the peak in rates of molecular and phenotypic evolution but the subsequent precipitous decline to background rates. Lee *et al.* (*27*) argued convincingly that this was unlikely to be an artifact of oversampling of characters changing on early arthropod branches because (i) they had selected phenotypic characters that changed at all hierarchies within their arthropod phylogeny and (ii) the pattern was replicated in the molecular dataset, which could not be biased in such a way. However, the marked reduction in the evolutionary rate may have a more prosaic explanation in that in all of these studies, the sampling of lineages is targeted to resolving branching relationships deep within animal phylogeny at the class level or among much more inclusive clades. A lineage-through-time plot (fig. S4), together with the accompanying temporal summary statistics (table S1), demonstrates that most of sampled lineages occur within an interval between 600 and 500 Ma (from 4.6 to 67.7%), approaching an asymptote at about 400 Ma (86.2%). Although lineage-through-time plots do not provide reliable estimates of diversification history under sparse taxon sampling (*34*), they summarize the temporal distribution of sampled lineages represented in the dataset. This is problematic given that by nonuniformly sampling lineages across time, we cannot expect to have sampled rates across time with an equal resolution, with lower rates expected from younger, effectively longer lineages across which rate changes are averaged and character changes are underestimated. Thus, while we consider the inference of increasing rates of morphological and molecular evolution to be accurate for early animals up until about 500 to 400 Ma, we have no confidence in the apparent deceleration that follows.

### Cambrian explosion: Whence and whither?

A number of hypotheses have been proposed to explain a Cambrian explosion of animals. We have attempted to characterize these hypotheses using nonmutually exclusive subsets of our phenotypic dataset. These partitions of traits, grouped into characters related to development/reproduction, feeding, locomotion, nervous system, and fossilization potential, exhibit distinct temporal and phylogenetic patterns. Traits associated with development and reproduction exhibit the earliest increase in rate in the middle Ediacaran, remaining elevated until the late Ediacaran (Fig. 2A), associated principally with the metazoan stem lineage and the nephrozoan crown (Fig. 3A), while feeding and fossilization show a middle Ediacaran rise and latest Ediacaran-earliest Cambrian peak in rate (Fig. 2, B and C), associated with stem-Bilateria, crown-Protostomia, and annelids (Fig. 3, B and C). Nervous system characters also exhibit a middle Ediacaran rise in evolutionary rate but peak within the late Ediacaran (Fig. 2E), associated with disparate branches, while traits associated with locomotion show a later rise and peak in the latest Ediacaran-earliest Cambrian (Fig. 2D), associated principally with stem-Bilateria and stem-Lophotrochozoa (Fig. 3D). Although the precise timing of peak rates differs among partitions, most elevated evolutionary rates across the Ediacaran-Cambrian transition do not help in discriminating among competing explanatory hypotheses. If locomotion- and feeding-related characters can be used to characterize the substrate and agronomic revolutions (*22*, *26*), then the inferred increases in the rate of evolution of associated phenotypic characters may fit with contemporaneously increasing styles and intensity of bioturbation, but the agronomic/substrate revolution proper is a Cambrian phenomenon (*2*, *22*).

Developmental characters exhibiting much earlier increases in evolutionary rate are perhaps compatible with the expectation that regulatory evolution must precede phenotypic diversification (*35*, *36*). This signal is particularly noteworthy because elevated developmental rates occur along relatively long and phylogenetically deep branches, especially the metazoan stem lineage, implying a sustained period of phenotypic change rather than a burst of novelty. The metazoan stem lineage exhibits one of the highest rates for the phenotypic dataset, yet among the five functional character partitions, only developmental (including reproduction-related) characters show similarly elevated rates on this branch. This suggests that developmental characters may contribute disproportionately to the overall peak in phenotypic evolution and may therefore represent a particularly important component of early animal evolutionary novelty. Fossilization-related traits suggest elevated rates associated with stem-Bilateria and crown-Protostomia (Fig. 3C) in a middle-end Ediacaran interval (Fig. 2C), in which there is a dearth of skeletal fossils but plenty of macrofossils with the fossilization-related traits. The pattern associated with nervous system traits, characterizing the purported “information revolution” (*23*, *24*), is perhaps the least compatible with the paleontological evidence. Although nervous system characters exhibit very high estimated rates on a small number of branches, these are concentrated on relatively short branches, where rate estimates are inherently more uncertain. Thus, while the observed peak is notable, it may not imply the same magnitude or duration of phenotypic change associated with the developmental/reproductive characters.

The temporal lag between the expectation of hypotheses and the rates of the evolution seen in associated phenotypic characters could be a product of uncertainties associated with the underlying evolutionary timescales or insufficiencies in the trait data. However, the temporal mismatch between hypothesized ecological impact and shifts in the evolutionary rate of associated phenotypic characters must occur necessarily because the phenotypic characters must evolve before they can have an ecological effect. This reflects the distinction between the rise of evolutionary novelties and their realization as evolutionary innovations, which are often temporally disassociated (*37*, *38*).

Our analyses do not support the hypothesis of a Cambrian explosion in evolutionary rate. The foundational changes associated with early animal evolution appear to have occurred in the middle Ediacaran to earliest Cambrian, associated with the establishment of the fundamental animal lineages, from the crown metazoan to the origin of animal phyla. Increased taxonomic richness and ecological diversity, which were precipitated by the bilaterian phyla after their Ediacaran-Cambrian establishment, are later phenomena (*11*).

There have been calls for the abandonment of the concept of a Cambrian explosion because there is no good evidence that it represents anything more than a diversification event (*39*). Our analyses indicate that elevated rates of molecular and phenotypic evolution originated during the Ediacaran and reached their highest levels around the Ediacaran-Cambrian transition, rather than representing a uniquely Cambrian phenomenon. Together, these data suggest a reformulation of our understanding of early metazoan evolution, precipitating late Ediacaran-early Cambrian explosions in ecology and diversity, which represent evolutionary innovations built on an earlier Ediacaran explosion of evolutionary novelty.

An important implication of our results is that both molecular and phenotypic rate accelerations are comparatively modest. Even under the most compressed evolutionary timescales, peak rates generally represent approximately an order-of-magnitude increase above background levels, rather than the extreme accelerations often implied by traditional interpretations of an evolutionary “explosion.” Similar conclusions have been reached in a previous relaxed-clock study (*27*), suggesting that the emergence of animal body plans may not have required exceptional evolutionary rates but rather sustained periods of elevated evolutionary change operating over tens of millions of years.

## MATERIALS AND METHODS

### Experimental design

We quantified and compared branch-specific molecular and phenotypic evolutionary rates across early animal phylogeny to test whether molecular acceleration preceded phenotypic diversification during the origin of major animal body plans. We integrated a large discrete morphological character matrix with a calibrated molecular phylogeny and estimated branch-wise relative rates under Bayesian relaxed-clock frameworks. To assess the robustness of temporal trajectories, we applied three alternative calibration strategies spanning Tonian-Cryogenian to late Ediacaran scenarios. We further partitioned the morphological matrix into functional character subsets to evaluate whether distinct biological systems exhibited staggered evolutionary pulses through time.

### Morphological dataset and phylogenetic framework

The morphological dataset comprised 2232 discrete characters scored across 65 taxa representing 16 major animal phyla. Of these, 1788 were compiled from previously published datasets (*28*), with an additional new 444 characters derived from reexamination of the original and newly incorporated source material. Characters were mostly coded as binary presence-absence statements (0 = absent; 1 = present) and scored for each taxon according to the condition specified in the character statement. To facilitate interpretation and traceability, the complete morphological matrix is available in the Figshare repository.

The phylogenetic topology was constrained using the calibrated molecular phylogeny of Carlisle *et al.* (*11*). To integrate the topology with the morphological matrix, terminal taxa were mapped to equivalent taxonomic units represented in the discrete character matrix, in some cases at higher clade levels. Taxa lacking direct correspondence between the two datasets were excluded from downstream morphological relaxed-clock analyses.

### Molecular evolutionary rate estimation

Branch-specific molecular substitution rates were estimated in MCMCtree from PAML (version 4.9) (*29*). We analyzed 66-taxon 30,031 amino acid positions comprising 203 nuclear protein–coding genes under the LG+Γ model (*11*, *12*) using the approximate-likelihood framework implemented for large phylogenomic datasets. The 203 nuclear protein–coding genes encompass a broad sample of the genomic sampling, but we do not argue that these data are representative of the genome as a whole. We did not use MrBayes to estimate rates of molecular evolution because projected runtimes were excessive even with the topology fully fixed and therefore difficult to justify on environmental grounds.

Following Carlisle *et al.* (*11*), the primary molecular analyses used an autocorrelated relaxed-clock model with soft-bound node calibrations and a uniform calibration-density scheme. The primary analyses used a uniform calibration-density scheme spanning the minimum and maximum constraints. Complete calibration trees for all analyses are provided in the Supplementary Materials. The overall mean substitution rate of the main tree (Fig. 1A) was estimated at 0.1655 substitutions per site per Myr, and relative branch-specific rates were calculated by dividing each posterior branch rate by this mean base rate.

The rate-variance prior (σ^2^) was specified as Γ (1,10), allowing substantial deviation from strict-clock expectations. Each analysis was run with a burn-in of 100,000 iterations, after which 20,000 posterior samples were collected at intervals of 1000 iterations. The analyses were run for a total of 20.1 million iterations. We ran five independent chains and assessed convergence using effective sample sizes (ESSs > 200) and consistency of posterior means across chains. The molecular analyses follow the preferred framework of Carlisle *et al.* (*11*).

To improve methodological comparability with the morphological rate analyses, we additionally analyzed the molecular dataset using an independent-rate model in MCMCtree. Branch-specific molecular rates inferred under the model were compared with morphological rates and used to reconstruct temporal rate trajectories under the alternative calibration schemes. Details and results are provided in figs. S6 to S8.

### Alternative calibration strategies

To test the sensitivity of inferred temporal rate trajectories to divergence-time assumptions, we implemented three alternative calibration schemes following previous studies (*11*, *12*, *17*):

1) Carlisle strategy using the original 45 soft-bound node calibration framework of Carlisle *et al.* (*11*).

2) dos Reis strategy using 34 calibrations following dos Reis *et al.* (*12*).

3) Budd and Mann strategy in which four critical deep nodes were recalibrated using revised late Ediacaran ages from Budd and Mann (*17*), while all remaining nodes retained the priors from Carlisle *et al.* (*11*).

The complete calibration topologies used for each framework are provided in the Figshare repository.

### Phenotypic evolutionary rate estimation

Phenotypic evolutionary rates were estimated from the morphological matrix alone using MrBayes (version 3.2.7a) (*40*) under a relaxed morphological clock. The phylogenetic topology and node-age constraints followed the calibrated framework described above.

We implemented a uniform clock branch-length prior with independent gamma rates to model branch-specific variation. The overall clock-rate prior was specified as a lognormal distribution, clockratepr = lognorm(−6, 0.6), following previous morphological clock analyses (*11*)*.* The prior mean was chosen to reflect an expected number of character-state changes across a tree of the estimated depth and temporal duration, thereby providing a biologically reasonable scale for rate estimation, while the variance allowed substantial uncertainty around this expectation.

Two independent runs of four chains each were performed, each consisting of one cold chain and three heated chains (temperature parameter = 0.1), for 20 million generations, sampling every 5000 generations. The first 25% of samples were discarded as burn-in. Convergence and adequate mixing were confirmed using average split frequencies and ESS values exceeding 200. Posterior branch-wise rates were expressed as relative multipliers of the inferred mean morphological clock rate.

### Functional partitioning of morphological characters

To evaluate whether distinct biological themes exhibited different temporal evolutionary dynamics, we extracted targeted, biologically informed subsets of characters from the full morphological matrix. These subsets represented nervous system (299 characters), feeding (439 characters), locomotion (377 characters), fossilization potential (942 characters), and development and reproduction (564 characters). Characters were assigned to these categories manually on the basis of our interpretation of their biology; this categorization cannot be done through statistical optimization of partitions using methods like model fitting. As individual characters may be relevant to more than one category, overlapping membership was permitted.

The nervous system partition included sensory and neural characters. Feeding characters captured diverse trophic adaptations across metazoan clades. Locomotory characters included structures associated with active movement, such as musculature, appendages, parapodia, chaetae, and swimming or walking adaptations. Fossilization-related traits followed the taphonomic character definitions of Carlisle *et al.* (*11*). Developmental-reproductive characters included traits related to cell differentiation, reproductive mode, and larval development. Each partition was analyzed independently using the same relaxed-clock framework as the full phenotypic dataset.

### Statistical analysis

We summarized temporal variation in branch-specific relative rates using LOESS regression of rate against node age, with 95% confidence intervals estimated from the fitted model. This nonparametric framework was selected to capture the nonlinear temporal structure without imposing a predefined functional form (*41*).

To identify branches exhibiting markedly accelerated and decelerated rates, we compared each branch-specific rate (Δ*v*) to the empirical background distribution of all branch rates across the tree. Branches were classified as accelerated when Δ*v* > μΔ*b* + σ and decelerated when Δ*v* < μΔ*b* − σ, where μΔ*b* and σ represent the mean and standard deviation of the background rate distribution, respectively. This empirical thresholding was used as a descriptive criterion to identify branches exhibiting rates substantially different from the overall background distribution. Because molecular and phenotypic rates are measured on different scales, application of the same empirical thresholding criterion (μ + σ) yielded different dataset-specific thresholds (1.13 for overall molecular rates and 12.10 for overall phenotypic rates). This empirical thresholding approach follows the conceptual frameworks of Baker *et al.* (*42*) in which branch-specific rates are evaluated relative to a background rate to identify branches exhibiting unusually high rates

### CCF and ARIMAX analyses

To further examine temporal relationships between molecular and morphological evolutionary rates, we conducted exploratory time-series analyses using temporally binned branch-rate estimates. Branches were assigned to 10-Myr time bins on the basis of their mean branch ages. Within each bin, we calculated the mean log-transformed morphological rate and mean log-transformed molecular rate. The number of branches contributing to each bin was also recorded to assess the support for each temporal estimate. The resulting binned rate series were ordered from older to younger time bins and converted into time-series objects in R. We first calculated the raw CCF between the morphological and molecular rate series to assess whether the two rate trajectories showed correlated variation at different temporal lags (*43*). Because raw CCFs can be influenced by autocorrelation and the shared temporal structure, we also calculated a prewhitened CCF. For this analysis, an ARIMA model was fitted to the molecular rate series using auto.arima from the R package forecast (*44*). The residuals of this model were used as the prewhitened molecular series, and the same ARIMA filter was then applied to the morphological rate series. The CCF was then calculated between the filtered morphological series and the residual molecular series. We also fitted an ARIMAX model to test whether molecular rates explained variation in morphological rates after accounting for the temporal structure in the response series (*43*). In this model, the binned log morphological rate series was treated as the response variable, and the binned log molecular rate series was included as an external regressor. A null ARIMA model without the molecular-rate predictor was also fitted to the morphological rate series. Model support was compared using AIC, AICc (corrected AIC), and BIC (Bayesian information criterion), and the significance of the molecular-rate coefficient was assessed using the estimated coefficient, SE, *z* statistic, and associated two-tailed *P* value. These analyses were treated as exploratory because the time series was derived from binned branch-level estimates rather than from independently sampled temporal observations and because some time bins were represented by relatively few branches.
