# Supplementary material for: Metazoan diversification characterized by elevated rates of evolution during the middle Ediacaran to earliest Cambrian
Source: Sci Adv. 2026 Oct 30;12(44):eaeh7099. doi: 10.1126/sciadv.aeh7099 (PMC13721979; doi:10.1126/sciadv.aeh7099)
Supplement: Supplementary file 1 [file sciadv.aeh7099_sm.pdf]

Science Advances
AAAS

Supplementary Materials for

Metazoan diversification characterized by elevated rates of evolution during the middle Ediacaran to earliest Cambrian

Zhiqing Guo *et al.*

Corresponding author: Zhiqing Guo, zhiqing.guo@bristol.ac.uk;
Philip C. J. Donoghue, phil.donoghue@bristol.ac.uk

Sci. Adv. **12**, eaeh7099 (2026)
DOI: 10.1126/sciadv.aeh7099

This PDF file includes:

Figs. S1 to S8
Tables S1 to S4

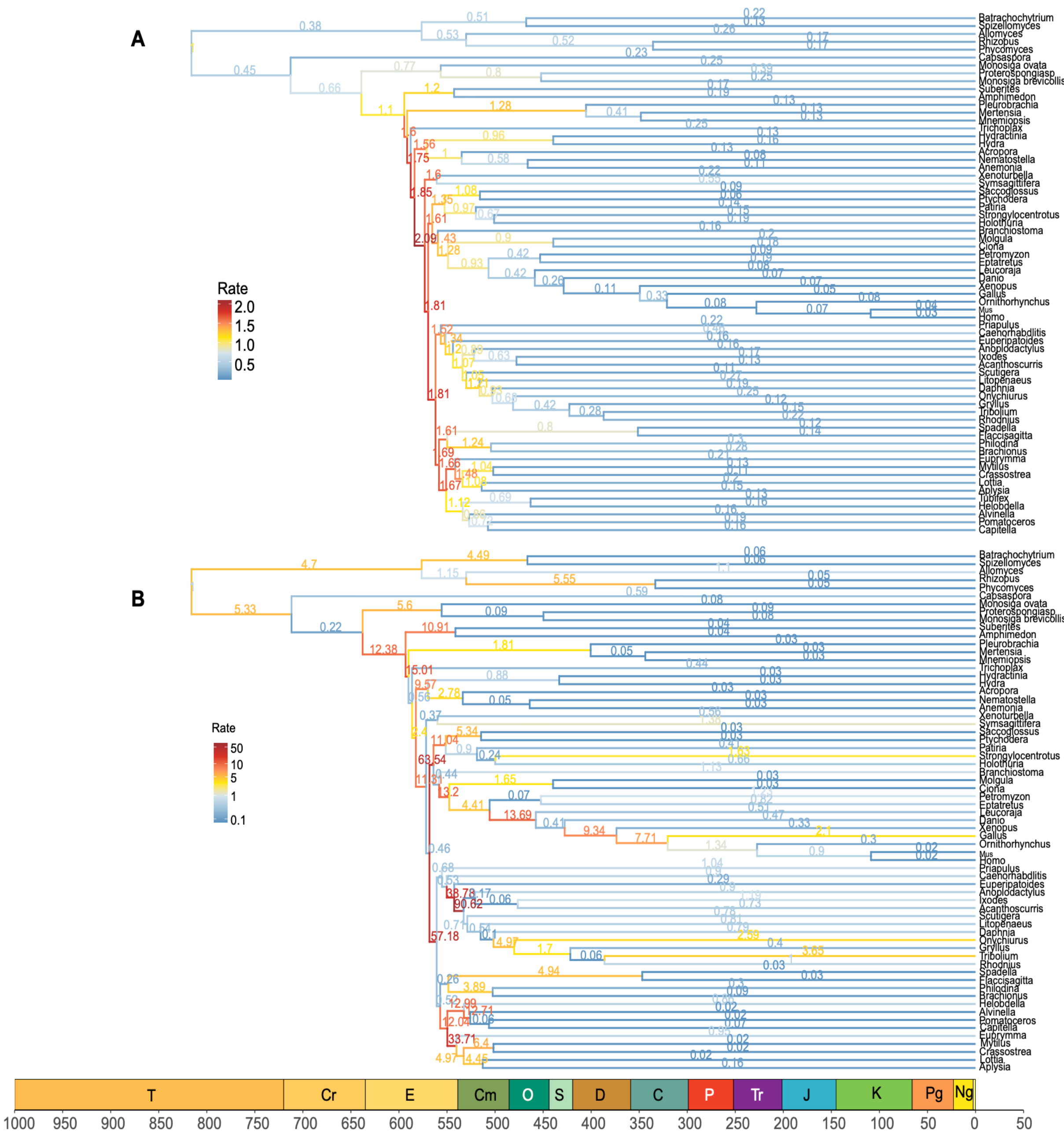

Fig. S1. The overall Evolutionary rate.

(A) Morphological evolutionary rates. (B) Molecular evolutionary rates.

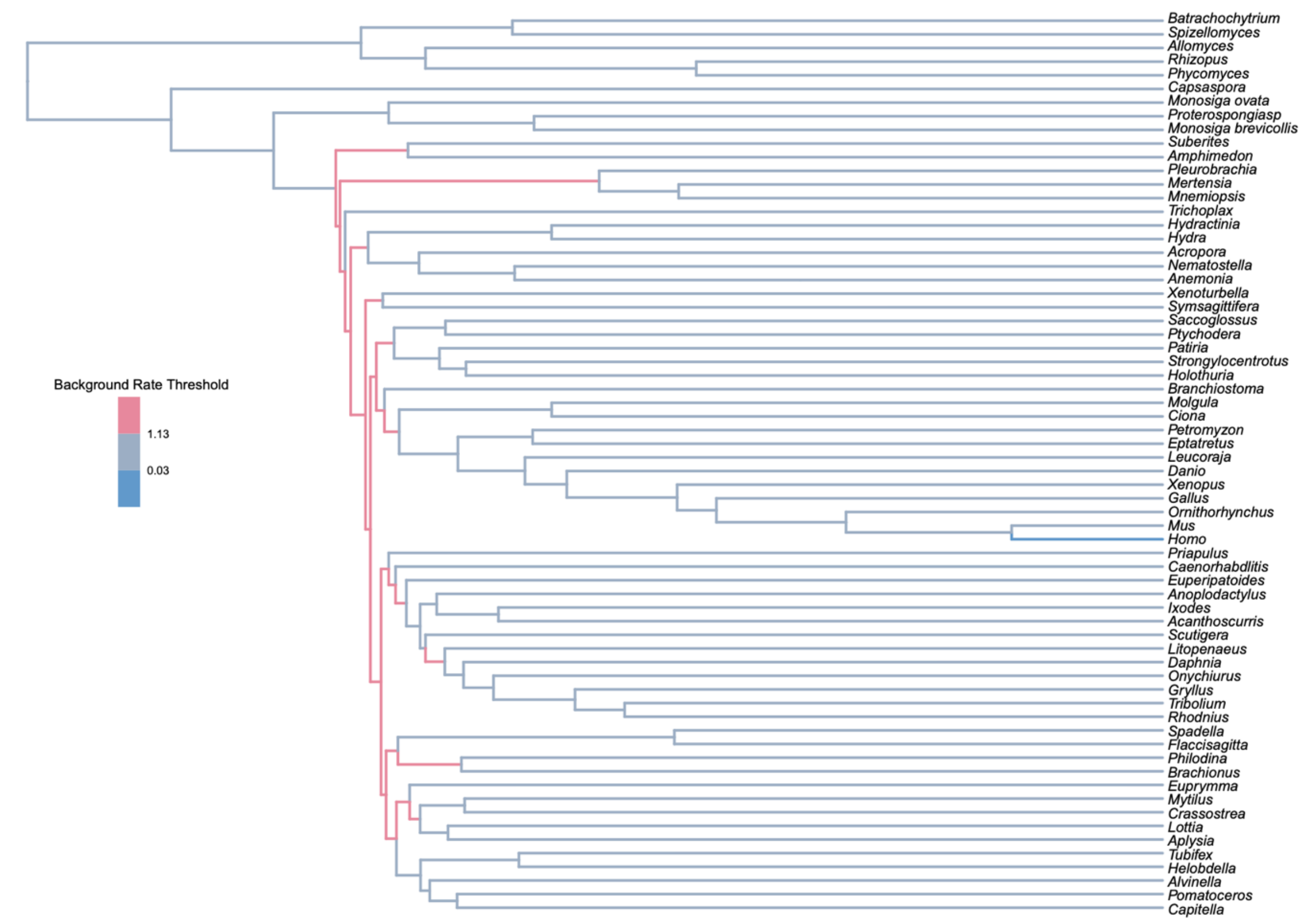

Fig. S2. Branches showing significant molecular rate shifts are highlighted.

Significant increases (rates > 1.13) are colored pink, significant decreases (rates < 0.03) are colored dark blue, and branches with non-significant rate shifts are colored grey-blue.

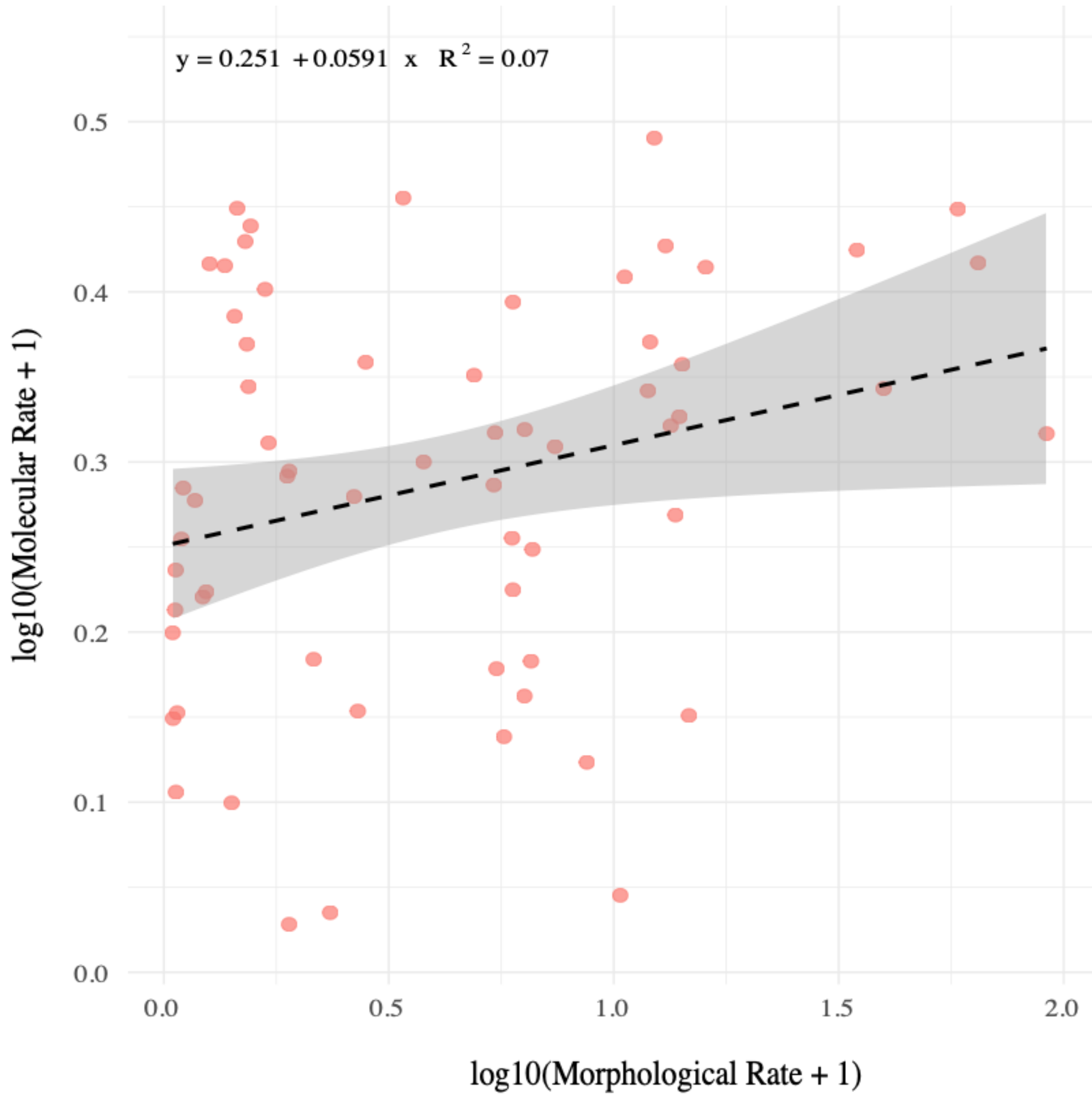

Fig. S3. Correlation between morphological and molecular evolutionary rates.

Log10-transformed morphological rates (x-axis) are plotted against log10-transformed molecular rates (y-axis). Rates were transformed by adding 1 prior to log-transformation (log10(rate+1)) to accommodate zero or near-zero values. The black dashed line represents the linear regression fit ($y = 0.251 + 0.0591x$, $R^2 = 0.07$).

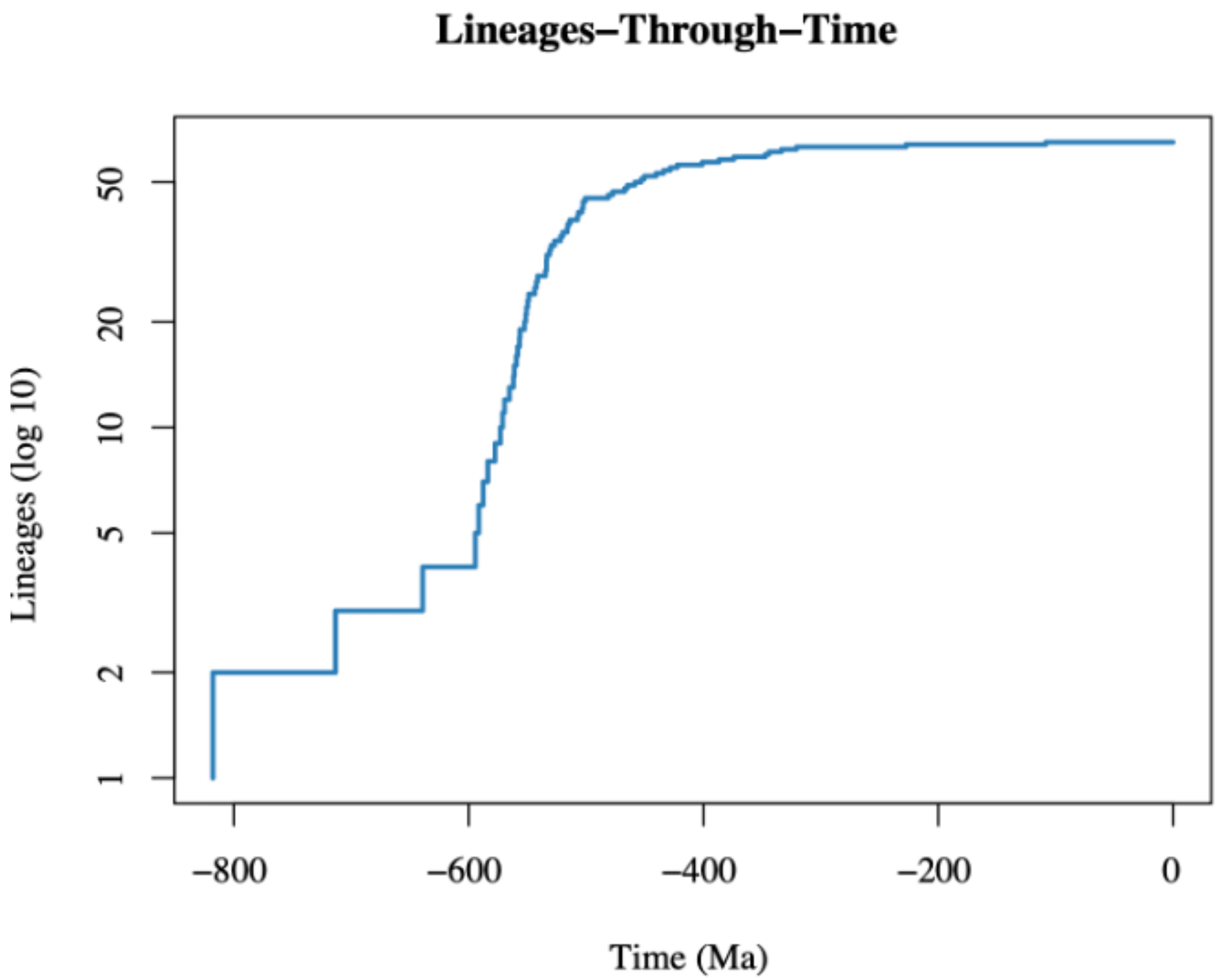

Fig. S4. Lineages-through-time (LTT) for the time-calibrated metazoan tree.

Cumulative number of lineages (log10) through absolute time (Ma; 0 = present). The curve shows a prolonged low accumulation before ~620 Ma. The LTT profile of the time-calibrated metazoan tree shows three phases. From 820 and 620 Ma the curve is nearly flat with small stepwise increments, indicating very low net lineage accumulation along deep branches. A marked inflection occurs near 610 and 590 Ma, followed by a steep rise through the latest Ediacaran into the early–middle Cambrian (about 540 to 505 Ma), during which the cumulative number of lineages on the log-scaled axis increases by roughly an order of magnitude. After 480 and 450 Ma, the trajectory approaches an asymptote at 51 sampled lineages, with only marginal additions towards the present.

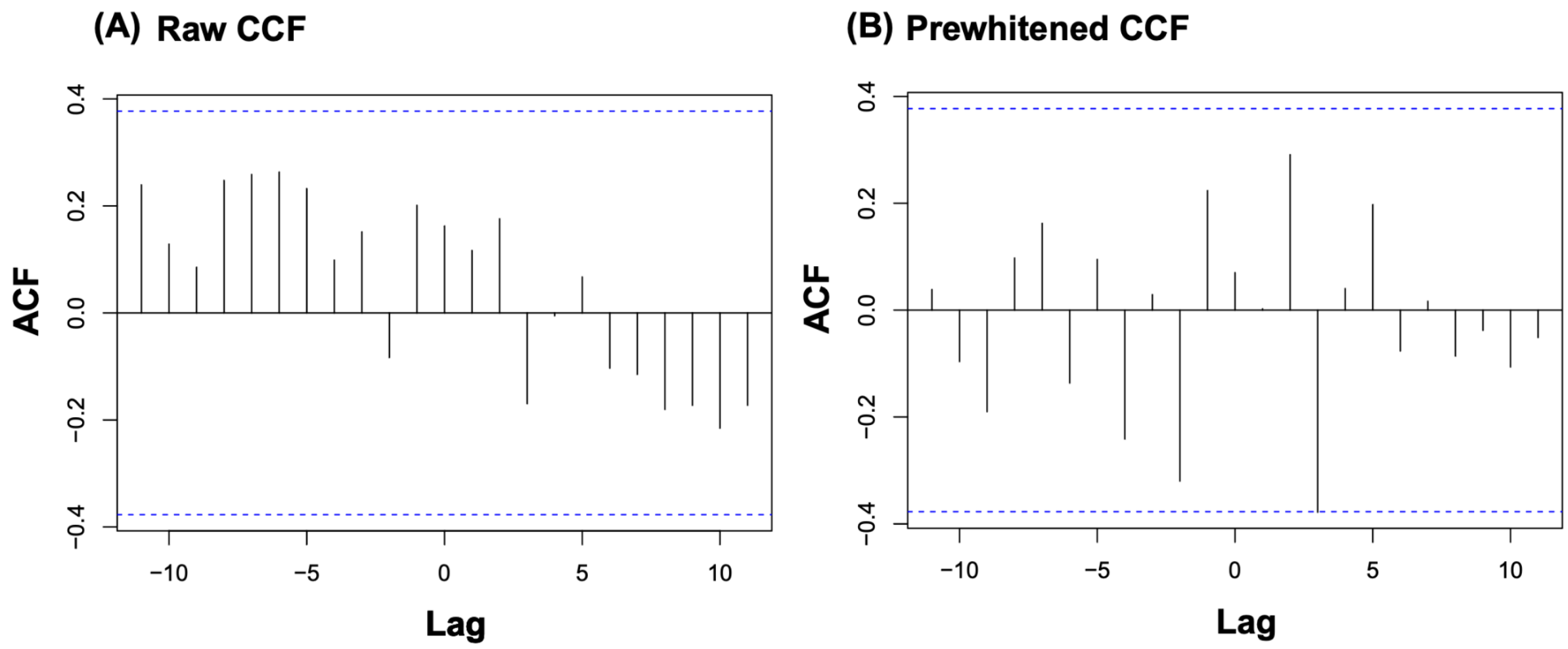

Fig. S5. Raw and Prewhitened cross-correlation function between binned molecular and morphological rates.

(**A**)Raw CCF calculated from 10 Myr-binned mean log morphological and molecular evolutionary rates. Lag units correspond to 10 Myr bins. (**B**)Prewhitened CCF calculated after fitting an ARIMA model to the molecular rate series and applying the same filter to the morphological rate series.

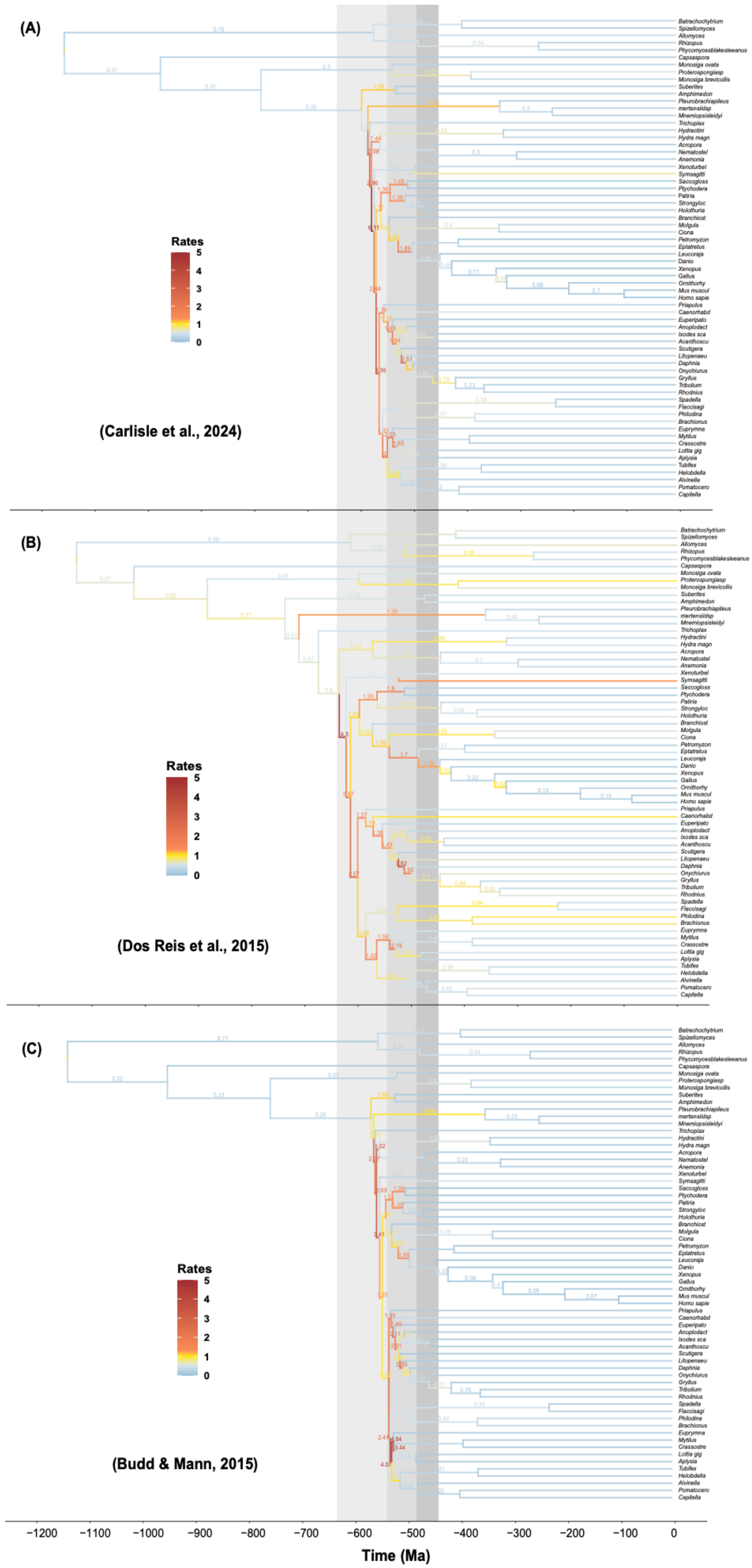
(A)
Rates
5
4
3
2
1
0
(Carlisle et al., 2024)
(B)
Rates
(Dos Reis et al., 2015)
(C)
Rates
(Budd & Mann, 2015)
−1200
−1100
−1000
−900
−800
−700
−600
−500
−400
−300
−200
−100
0
Time (Ma)

Fig. S6. Molecular evolutionary rates estimated under an independent-rates (IGR) relaxed-clock model across alternative divergence-time calibration strategies. (A) Carlisle et al. (10) calibration scheme; (B) dos Reis et al. (11) calibration scheme; (C) Budd & Mann calibration scheme (12). Branches are coloured according to log10-transformed molecular evolutionary rates (warmer colours indicate higher rates).

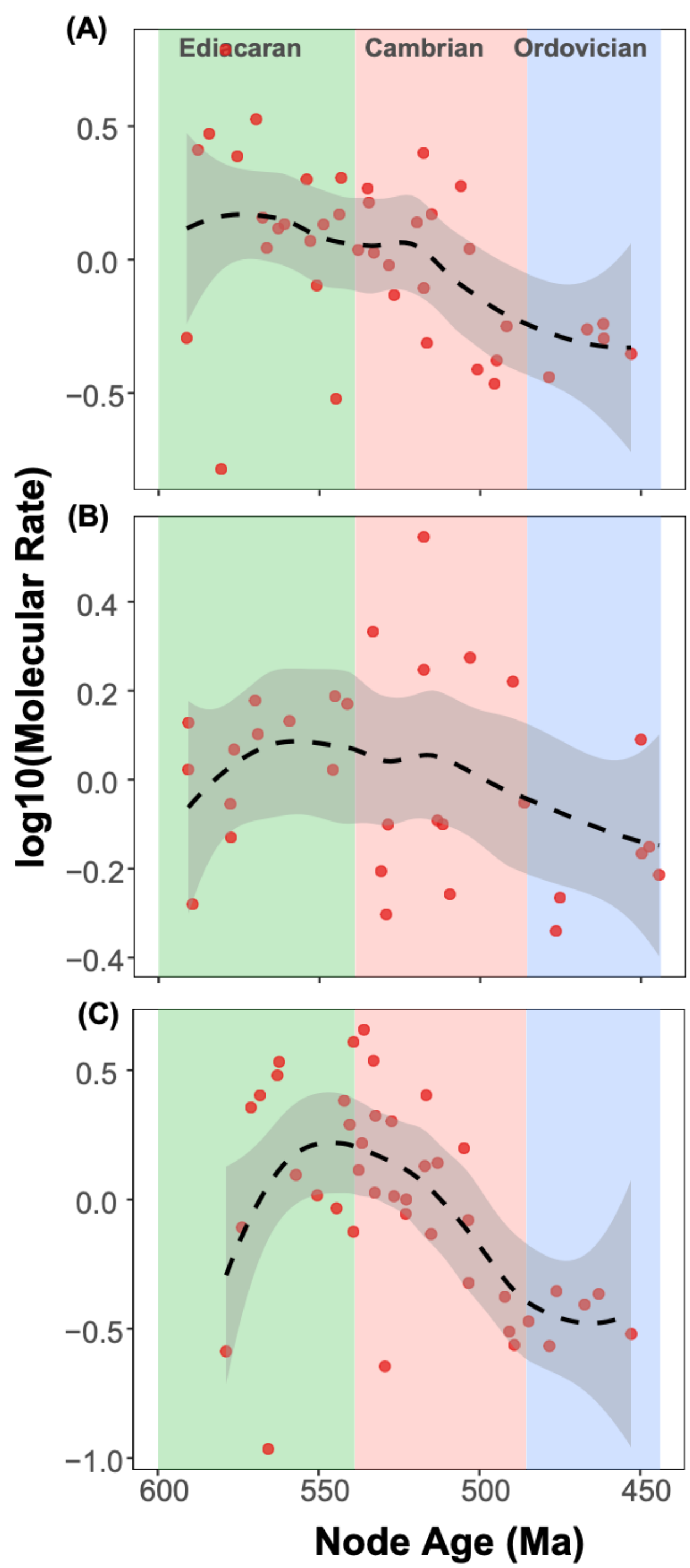

Fig. S7. Temporal trajectories of molecular evolutionary rates inferred under an independent-rates (IGR) relaxed-clock model across alternative divergence-time calibration strategies. (A) Carlisle et al. (10) calibration scheme; (B) dos Reis et al. (11) calibration scheme; (C) Budd & Mann calibration scheme (12). Branch-wise log10-transformed molecular rates are plotted against branch age (Ma), with LOESS smoothing curves (black dashed lines) and 95% confidence intervals (grey shading). Background colours indicate the Ediacaran, Cambrian, and Ordovician periods.

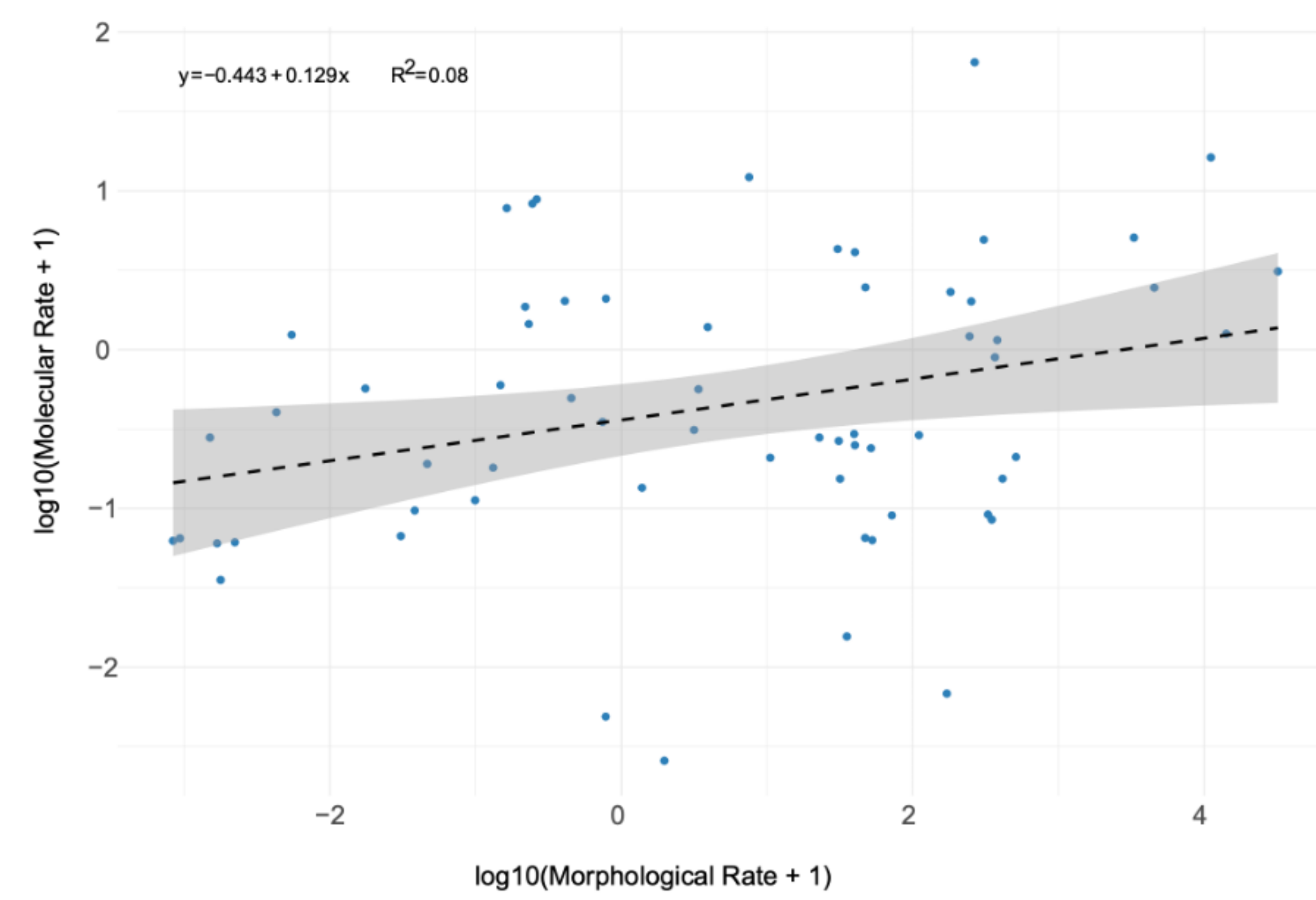

Fig. S8. Relationship between molecular and morphological evolutionary rates using molecular rates estimated under an independent-rates (IGR) relaxed-clock model. Branch-wise log10-transformed molecular rates are plotted against log10-transformed morphological rates. The dashed line shows the fitted linear regression and the grey shading indicates the 95% confidence interval. A weak positive relationship was recovered ($R^2 = 0.08$), indicating that variation in morphological rates explains only a small proportion of variation in molecular rates.

Table S1. Lineage-through-time summary statistics for sampled early animal lineages.

The table reports the number and percentage of sampled lineages present at successive temporal intervals, corresponding to the lineage-through-time profile shown in fig. S4.

**n_total**	**65**	
**age (Ma)**	**lineages_number**	**percent**
600	3	4.6
500	44	67.7
450	51	78.5
400	56	86.2

Table S2. Temporally binned molecular and morphological rate data used for CCF and ARIMAX analyses.
Branch-level molecular and morphological rates were assigned to 10 Myr bins based on mean branch age. For each bin, mean log-transformed morphological rate, mean log-transformed molecular rate, and the number of contributing branches are reported.

time_bin	log(morphological rates)	log(molecular rates)	n_branches
710	1.6732533	-0.7911716	1
630	-1.5140505	-0.4123324	1
590	2.61226643	0.27954008	2
580	0.14776156	0.58699732	2
570	2.07734187	0.06857767	3
560	1.60175711	0.53434269	4
550	0.58546486	0.30699956	7
540	2.16254182	0.31824254	5
530	1.96744417	-0.0138015	5
520	0.14716808	-0.0733233	3
510	0.61247461	0.07872844	4
500	-0.292876	-0.1015067	6

480	1.60279006	-0.3885444	1
470	-2.824101	-0.4573136	1
460	-0.788146	-0.6079214	2
450	-0.8015235	-0.6566067	3
440	0.49851091	-0.1009481	1
430	-0.1290352	-0.0439056	1
420	-0.1756855	-1.1068407	2
400	0.59289471	0.24982424	1
380	-2.7506707	-1.2870201	1
370	2.2341723	-2.2096586	1
340	-0.7162959	-0.5575679	2
330	1.71341083	-0.6466662	1
320	2.04300445	-1.1123976	1
220	0.2948456	-2.4756622	1
100	-0.1074193	-2.7027614	1

Table S3: ARIMA and ARIMAX model comparison for binned morphological and molecular rates.
The null ARIMA model used binned log morphological rate as the response without molecular rate as a predictor. The ARIMAX model included binned log molecular rate as an external regressor. Model support was compared using AIC, AICc, and BIC. Lower AIC values indicate stronger model support.

Model	Response	Predictor	N bins	Error structure	Estimate	SE	z	p	AIC	AICc	BIC
Null ARIMA	log morph rate	none	27	ARIMA(0,0,2)	—	—	—	—	98.94	99.98	102.82
ARIMAX	log morph rate	log mol rate	27	ARIMA(0,0,0)	0.273	0.319	0.855	0.393	101.20	102.24	105.08

Table S4. ARIMA model used for prewhitening the molecular rate series.
The molecular rate series was modelled using ARIMA(0,1,1), and the resulting residuals were used for the prewhitened CCF analysis.

lag	Raw_correlation	Prewhitened_correlation
-11	0.23942044	0.03847037
-10	0.128490806	-0.0961783
-9	0.085331162	-0.1899733
-8	0.247771574	0.09747936
-7	0.258777981	0.16243782
-6	0.263306434	-0.1363308
-5	0.232353634	0.09480826
-4	0.098713442	-0.2412081
-3	0.15143292	0.02883407
-2	-0.083365567	-0.3198168
-1	0.201134079	0.22361765

0	0.162289042	0.07005549
1	0.116651092	0.00231388
2	0.175919817	0.29078455
3	-0.169388575	-0.3781768
4	-0.005379673	0.04032756
5	0.067284684	0.19744364
6	-0.103089827	-0.0764093
7	-0.114801061	0.01658499
8	-0.180424339	-0.0858504
9	-0.172689409	-0.0376129
10	-0.215406553	-0.1061803
11	-0.172620059	-0.0510933
